# Rumen metagenome profiles are heritable and rank the New Zealand national sheep flock for enteric methane emissions

**DOI:** 10.1186/s12711-025-00973-3

**Published:** 2025-05-27

**Authors:** Timothy P. Bilton, Setegn W. Alemu, Ken G. Dodds, Hannah Henry, Melanie K. Hess, Ronan Jordan, Fern Booker, Sharon M. Hickey, Neville Amyes, Kevin Knowler, Edgar Sandoval, Jacqueline Peers-Adams, Tracey C. van Stijn, Hayley Baird, Trevor Watson, Wendy Bain, Barry Veenvliet, Gerard Pile, Brooke Bryson, Shannon M. Clarke, Patricia L. Johnson, John C. McEwan, Suzanne J. Rowe

**Affiliations:** 1 AgResearch, Invermay Agricultural Centre, Private Bag 50034, Mosgiel, 9053 New Zealand; 2 AgResearch, Ruakura Research Centre, Private Bag 3123, Hamilton, 3240 New Zealand; 3 AgResearch, Grasslands Research Centre, Private Bag 11008, Palmerston North, 4442 New Zealand; 4 AgResearch, Woodlands Research Farm, RD1, Woodlands, Invercargill, 9871 New Zealand

## Abstract

**Background:**

Global targets to reduce greenhouse gas emissions to meet international climate change commitments have driven the livestock industry to develop solutions to reduce methane emission in ruminants while maintaining production. Research has shown that selective breeding for low methane emitting ruminants using genomic selection is one viable solution to meet methane targets at a national level. However, this requires obtaining sufficient measures of methane on individual animals across the national herd. In sheep, one affordable method for measuring methane on-farm to rank animals on their methane emissions is portable accumulation chambers (PAC), although this method is not without its challenges. An alternative is to use a proxy trait that is genetically correlated with PAC methane measures. One such trait that has shown promise is rumen metagenome community (RMC) profiles. In this study, we investigate the potential of using RMC profiles as a proxy trait for methane emissions from PAC using a large sheep dataset consisting of 4585 mixed-sex lambs from several flocks and years across New Zealand.

**Results:**

RMC profiles were generated from rumen samples collected on the animals immediately after being measured through PAC using restriction enzyme-reduced representation sequencing. We predicted methane (CH_4_) and carbon dioxide (CO_2_) emissions (grams per day), as well as the ratio CH_4_/(CO_2_ + CH_4_) (CH_4_Ratio), from the RMC profiles and SNP-array genotype data. Heritability and microbiability estimates were similar to values found in the literature for all traits. The correlation of PAC methane with predicted methane was 1.9- to 2.3-fold (CH_4_) and 1.2- to 1.5-fold (CH_4_Ratio) greater for RMC profiles compared to host genomics only. The genetic correlation between methane predicted from RMC profiles and PAC methane was 0.75 ± 0.12 for CH_4_ and 0.64 ± 0.11 for CH_4_Ratio when using a validation set consisting of the animals with the most recent year of birth in the dataset.

**Conclusions:**

RMC profiles are predictive of, and genetically correlated, with PAC methane measures. Therefore, RMC profiles are a suitable proxy trait for determining the genetic merit of an animal’s methane emissions and could be incorporated into existing breeding programs to facilitate selective breeding for low methane emitting sheep.

**Supplementary Information:**

The online version contains supplementary material available at 10.1186/s12711-025-00973-3.

## Background

In the wake of pressing global environmental challenges, targets for mitigating the adverse effects of climate change have been clearly delineated by prominent local and international organizations and governments. Part of this mitigation strategy is the reduction of enteric methane from ruminants, which is estimated to account for approximately 6% of the global anthropogenic greenhouse gas emissions [1]. Consequently, commitments to reduce methane emissions by 30% by 2030 relative to 2020 levels have been made by over 50 countries as part of an attempt to mitigate against the impacts of climate change [2]. In New Zealand, enteric methane emissions from ruminants is estimated to be responsible for 73.7% of the total emissions from the agricultural sector and 36.3% of the gross emissions [3]. Methane reduction targets of 10% by 2030 and between 24 and 47% by 2050 relative to biogenic methane emission levels in 2017 have recently been set by the New Zealand government [4]. However, ruminants play an important role in feeding the world for food security and combating poverty [5, 6]. Hence, it is vital that reductions in methane emissions can be achieved without compromising production to secure the future of the agricultural industry. To address these challenges, new technologies and innovative solutions will be required [6].

One strategy towards achieving methane targets revolves around dietary alterations and feed additive integrations, which could modulate metabolic processes within ruminants’ digestive system that lead to diminished methane production [7]. Despite promising results, this approach demands continuous implementation of specific diets or additives, making it a long-term commitment [8]. Furthermore, this strategy must contend with the adaptability of rumen microbiomes [9], that constitute a diverse ecosystems of bacteria, protozoa, and fungi, and play a critical role in ruminants’ digestion [10]. These microbial communities are highly dynamic, adjusting to dietary shifts [11], adding a layer of complexity for dietary methane mitigation strategies.

The global challenge of reducing methane emissions from ruminants may benefit from solutions in the domain of host genetics. Genetic selection emerges as a viable, sustainable, and potentially long-lasting solution to this environmental challenge [12]. The essence of this approach hinges on the idea of leveraging the genetic variability in existing populations, that is to use the inherent genetic traits of ruminants, aiming for selective breeding to gradually decrease methane emissions over successive generations. Supporting this perspective is a series of research findings that emphasize the heritability of methane traits in ruminants. Studies spanning over a decade, from early work by Haas et al. [13] to more recent findings by Rowe et al. [14], have consistently underscored that ruminant methane emissions have a host genetic component. Such discoveries highlight the promise that genetic selection holds, where targeted breeding could result in generations of ruminants with naturally lower methane emissions.

A major challenge to breeding for lower methane emitting animals is obtaining reliable on-farm phenotypic measures of methane on a large scale that can be used to rank animals on their methane emissions for selection purposes. The most reliable method for measuring methane from ruminants is respiration chambers [15], but these are expensive, low-throughput and not practical for on-farm application. One often overlooked aspect of respiration chambers is that reported respiration chamber measurements are typically not fed ad-lib but on the basis of liveweight [16] and this means any phenotypic or genetic comparison with methane collected from ad-lib pasture grazing measurements need to take this into account [17]. Other technologies, such as the GreenFeed System (C-Lock Inc., Rapid City, SD) and the sulphur hexafluoride tracer method [18], are also available but are generally high cost, labour intensive and low-throughout which inhibits the ability to measure large number of animals. Sniffers provide an on-farm, high-throughput method for measuring methane at automatic milking stations or feeder bins but requires a large number of measurements for a reliable estimate of methane as sniffer measurements are quite variable [19]. For sheep, an alternative technology that has gained rapid adoption in recent years is portable accumulation chambers (PAC) [16, 20] that collects methane, oxygen, and carbon dioxide gas measurements of individual sheep while in a gas chamber for approximately an hour. PAC provides an affordable and portable method for measuring methane on-farm and its 1-h measure of methane related traits is sufficient for breeding purposes. A previous study by Robinson et al. [17] has shown PAC to have a moderately-to-high repeatability and genetic correlation with respiration chamber measurements indicating the suitability of PAC for breeding. PAC has also proven very successful for obtaining commercial measures of enteric methane with a large uptake in adoption by breeders and farms in recent years, where in New Zealand over 5000 commercial measures are being collected per year [21]. However, there are some challenges with using PAC. One challenge is logistics as the number of PAC units available is insufficient to meet demand from breeders who are often wanting to measure their flocks at similar times of the year. Another limitation of PAC is that it does not provide information on individual feed intakes, which is important for breeding for lower methane emitting sheep.

An alternative approach to using PAC would be to obtain an indirect measure of PAC methane emissions via a proxy trait, for the purpose of ranking animals on their methane emissions. One possibility would be to use rumen microbial communities or rumen metagenome communities (RMC) profiles to predict PAC methane emissions, whereby predictions from the RMC data forms the proxy trait used to rank animals on their PAC methane emissions. In ruminants, methane is produced as a by-product of a complex microbial fermentation process in the rumen that converts feed into volatile fatty acids, which are then absorbed through the gut wall and are a major source of energy for the animal [22]. This process is controlled by the microorganisms in the rumen microbiome and therefore changes in the RMC profiles are likely associated with differences in enteric methane production in ruminants. To-date, studies have provided evidence that RMC profiles are both partially controlled by host genetics and are predictive of methane emissions in various ruminants [23–28]. Utilization of information from the RMC profiles requires an appropriate model to use this RMC information to predict methane or other traits of interest. Ross et al. [29] proposed the metagenome relationship matrix (MRM) to describe the relationships between RMC profiles between individuals and used this matrix in a conventional GBLUP type model where the genomic relationship matrix is replaced by the MRM to predict various traits of interest in humans and cattle from the RMC profiles. Several studies have utilized this approach on various species for a variety of traits, where different ways to construct the MRM have been developed [29–34]. For this study, we build on the work by Hess et al. [30] and utilize their model for RMC profiles which has been shown to have good performance for methane traits.

The aim of this study is to investigate the potential of using RMC profiles as a proxy trait for PAC methane to rank animals on their methane emissions for selective breeding. This requires determining the genetic and phenotype parameters of an RMC proxy trait for methane with PAC methane measures. A few studies have estimated the genetic correlation between some form of RMC methane trait with methane traits measured using various technologies and although the genetic correlations have been moderate to high, these studies have only had a relatively small number of animals [35, 36]. Here, we determine the genetic and phenotypic parameters of RMC proxy traits for methane with methane measured from PAC for sheep using a dataset consisting of over 4500 animals. To achieve this, we investigate the correlation of RMC profiles with PAC methane traits and then fit bivariate models to estimate the genetic and phenotypic correlations of the RMC proxy traits with the PAC methane traits.

## Methods

The animal experiments conducted adhered to the guidelines of the 1999 New Zealand Animal Welfare Act and AgResearch Code of Ethical Conduct. The research undertaken in this study, encompassing all experimental procedures and the handling of animals, was reviewed and subsequently approved by the AgResearch Animal Ethics committee (Ruakura, NZ). Approvals for our research protocols were granted under reference numbers: 13081, 13419, 13563, 13742, 13892, 14055, 14066, 14221, 14830, 14906, 14907, 14954, 14981, 15293, 15294, and 15439.

### Animals

This study leveraged data collected across two research flocks, three industry progeny test flocks and three industry performance-recorded sheep flocks located across New Zealand. The animals were a mixture of ram lambs (n = 2159) and ewe lambs (n = 2426) that were born over a span of 8 years, starting from 2014 and extending up to 2021 depending on the flock (Table 1). The animals born in 2014 to 2016 from flocks 1 to 3 consists of the Grass Lamb set of animals previously described and analysed by Hess et al. [23] and Hess et al. [30]. The flocks ranged from purebred animals (Romney and Perendale) from the industry performance-recorded sheep flocks through to composites. The composites were based on New Zealand maternal sheep breeds including Romney, Coopworth, Perendale, and Texel, with some minority maternal breeds such as Wiltshire and Finnish Landrace also contributing to the composite populations. Of note, one of the research flocks included in this study was the AgResearch Methane Selection line flock as described by Pinares-Patiño et al. [15].

**Table 1:** Animal numbers by flock, sex and year of birth

Flock	Year of Birth							Total
	2014	2015	2016	2018	2019	2020	2021	
Ewes								
1	142	152	159					453
2	65	149	149		101	117	323	904
3	95	96	57		76	135	136	595
4						236	90	326
5						148		148
Rams								
1				188	172	502	384	1246
3						125	128	253
6				161	168			329
7				81				81
8						250		250
Total	302	397	365	430	517	1513	1061	4585

### Phenotypes

All animals in this study were run through portable accumulation chamber (PAC) units to measure individual enteric methane emissions following the protocol outlined by Jonker et al. [16]. Methane measurements occurred when the animals were still young (between 3 and 14 months of age) and when the animals were off feed for at least 1 h and no more than 4 h after grazing ad-lib on pasture with sufficient levels of day-matter for at least 3 days. Animals were allocated and simultaneously measured in groups (or lots) of 12 using the PAC units, where allocations were made to balance for sire, and methane (CH_4_), carbon dioxide (CO_2_) and oxygen (O_2_) concentrations were recorded across a 50-min period. A small number of measurements were discarded due data quality issues (e.g., broken seal on chamber). The number of animals with reliable PAC measurements is given in Table 2. The three research flocks were measured using the PAC facility located at the AgResearch Woodlands research farm (Invercargill, NZ) whereas the five industry flocks were measured on-farm using the AgResearch PAC trailer.

**Table 2:** Number of records for each trait

Trait Group	Trait	Unit	Number of records
SIL	Liveweight 8 months (LW8)	kg	4574
Methane	Methane (CH_4_)	g/day	4532
	Methane ratio (CH_4_Ratio)	nM/Mol	4532
	Carbon dioxide (CO_2_)	g/day	4555
Rumen	Rumen metagenome		4585

The raw gas concentrations were transformed in grams per day (g/day) estimates using standard gas equations and the molar ratio trait CH_4_/(CO_2_ + CH_4_) (CH_4_Ratio) was calculated from molar CH_4_ and molar CO_2_ according to the protocol outlined by Jonker et al. [37], where the calculation equations are presented in Rodrigues et al. [38]. These traits were then scaled by dividing each individual value by the average value of the lot and multiplying by the overall population mean (7.5 g/day for CH_4_, 623 g/day for CO_2_, 0.032 for CH_4_Ratio). This scaling approach is used to (a) set the average methane emissions to be consistent across all lots and (b) stabilise the heterogenous variance of methane values. Variation in the average methane emissions across lots occurs primarily because the time animals have been off feed before entering the PAC units varies across the lots. Scaling is therefore required in order to appropriately rank the animals on their methane emissions from PAC. Note these scaling values, while expressed on a per 24-h basis, reflect the pre-measurement protocol and age of the animals and are not equivalent to expected 24-h emissions if the animals were freely grazing at pasture for 24 h. The traits evaluated in this study were the scaled CH_4_, scaled CO_2_ and the scaled ratio trait CH_4_Ratio.

Additional phenotype information was extracted from the Sheep Improvement Limited (SIL) database [39]. This included animal traits and contemporary group information to be used as fixed effects in the models. In addition, the SIL trait liveweight at 8 months (LW8) was extracted and is used as a benchmark for the analysis in this study.

### Rumen sampling and sequencing

All animals in this study were rumen sampled immediately after they were measured through the PAC units. Rumen samples were collected via stomach intubation and preserved either using a method of fast freezing on dry ice and freeze-dried (a.k.a., Frozen) as described by Kittelmann et al. [40] and modified by Hess et al. [41] or using the TNx2 preservative solution method developed by Budel et al. [42]. Rumen samples collected on animals born prior to 2020 were all preserved using the Frozen method whereas for the born 2020 and 2021 animals, all rumen samples were preserved using the TNx2 method. For both preservative methods, DNA from the rumen samples was extracted using a phenol–chloroform with bead beating protocol developed by Rius et al. [43] that was modified for each preservative method as previously described [40, 42]. The DNA extracted from the rumens samples was sequenced using the restriction enzyme-reduced representation sequencing (RE-RRS) approach described by Hess et al. [41] utilizing the restriction enzyme *Pst*I. Samples were processed into multiple libraries (consisting of four 96-well plates) and sequenced across multiple flowcells on either an Illumina HiSeq2500 (San Diego, CA, USA; 275 High Output Run mode, generating 101 bp single end reads using version 4 chemistry) or NovaSeq6000 (San Diego, CA, USA) with S1 or SP flowcells. RE-RRS was used instead of other sequencing approaches (such 16S or whole genome metagenomic sequencing) commonly used in metagenomic studies as it is a low-cost, high-throughput method that enables sequencing of large number of samples (> 1000). Previous research has shown that RE-RRS performs similarly or better than 16S sequencing [41] and allows application of a reference-free approach that facilities prediction of important livestock traits [30].

### Bioinformatic processing

Processing of the sequencing reads generated was performed using the same process outlined in Hess et al. [23]. Briefly, demultiplexing was performed using GBSX [44] and reads were trimmed using cutadapt [45] using a Phred quality score threshold of 20 and 40 bp for minimal length of individual reads. Samples were discarded if the total number reads remaining was less than 100,000. The reference-free (RF) pipeline was used to generate a RMC profile, which consists of a count matrix of tags (which are unique 65 bp trimmed reads starting from the initial cut site) representing different microbial genomes. Tags were only retained if they were present (i.e., at least one read) in at least 25% of the samples in a specified set of samples. The RF pipeline have previously been shown to generate an RMC profile that sufficiently describes the rumen microbiome [23].

A metagenome relationship matrix (MRM) was constructed from the RMC profiles using the following steps: (1) Adding a pseudo count of 1 to the RMC count matrix and convert into proportions by dividing by the total number of reads for the sample (row sums). (2) Transform the proportions using the log_10_ function. (3) Normalize each column of the transformed proportions matrix based on a “cohort” variable (defined as the combination of sequencing flowcell, flock code, year of birth and sex) such that each column has mean 0 and standard deviation 1 for each cohort. (4). The MRM is then generated by computing the correlation of the transposed cohort-normalized transformed proportion matrix, which effectively computes the correlation of the cohort-normalized RMC profiles between each pair of individuals. There were 38 cohorts in total with an average size of 93 ± 84 animals per cohort. All animals within a cohort were measured over a period spanning a maximum of three consecutive days and all samples within a cohort were preserved using the same method. The rationale behind using the cohort adjustment in the MRM is to remove environmental (e.g., sampling year, farms management, feed content) and laboratory variation that can introduce scale effects which obscures the signal in the RMC profile for the trait of interest (see Additional file 1, Fig. S1 which shows that there is no population structure in the MRM for this study). It is important to remove these scale effects as they can invalidate the implicit assumption of our modelling approach that animals rank similar across environments. It has been shown that using this cohort adjustment improves prediction accuracy [30]. Normalization of the RMC was also performed to ensure that each tag had equal weighting when constructing the MRM, as the effect of each tag on the trait of interest is not necessarily proportional to their relative abundance. It should be noted that the method we have used to generate the MRM ignores the compositionality of microbiome data. However, due to the large number of tags in the metagenome profile, the method we employ provides an approximation to using a traditional log-ratio transformation when constructing the MRM (see Additional file 2, Fig. S2). Furthermore, the approach we have used for constructing the MRM follows a similar rationale to constructing GRMs from SNP data, where similar assumptions are made (i.e., independent tags/SNPs, filter rare tags/SNPs). This approach to constructing the MRM is effectively employed in a number of animal genetics studies using rumen microbiome data found in the literature [30, 46–49].

### Animal genotyping

All animals used in this study were genotyped using one or more of 13 different SNP-arrays (> 5200 SNPs). All but 181 animals had genotypes from arrays with at least 15,000 SNPs. Filtering was performed such that only autosomal SNPs and SNPs with minor allele frequency greater than 0.01 and a call rate of 70% were retained, resulting in 14,325 SNPs in total with a mean call rate of 0.96. A genomic relationship matrix (GRM) was constructed using the KGD software [50] with Van Raden method 1 [51]. For each pair of individuals, only SNPs with non-missing genotypes were used to compute the genomic relationship. This assumes missingness at random and was computed efficiently using matrix operations in KGD.

### Statistical analysis

#### Genetic diversity

The diversity of the genetic relatedness of the animals in this study was benchmarked against 13,118 animals included in a study by Dodds et al. [52] investigating the breed composition of sheep across the New Zealand industry. A combined GRM was constructed using the animals from this study along with the 13,118 animals from the Dodds et al. [52] study. The GRM was constructed using the same methodology described earlier with 10,954 SNPs in common (missing rate was 0.99%) and 181 animals from this study were dropped due to having missing genotypes for more than 30% of the SNPs. The genetic relatedness was visualized by plotting the first two principal components (PC) from a principal component analysis of the combined GRM.

#### Microbiability and heritability

The proportion of variance explained by the host genomics (heritability) and by the RMC profile (microbiability) was estimated using the linear mixed modelling approach described in Hess et al. [30]. The models were fitted using ASReml v4.2 [53] and were of the form:

1 $${\mathbf{y}} = {\mathbf{Xb}} + {\mathbf{Zm}} + {\mathbf{e}}_{m}$$

2 $${\mathbf{y}} = {\mathbf{Xb}} + {\mathbf{Zg}} + {\mathbf{e}}_{g}$$

where $\mathbf{y}$ represents the trait of interest (LW8, CH_4_, CH_4_Ratio, or CO_2_), **X** is the incidence matrix for fixed effects, $\mathbf{b}$ is the vector of fixed effects, $\mathbf{Z}$ is the incidence matrix for the random effect, $\mathbf{m}$ is the vector of microbial effects, $\mathbf{g}$ is the vector of additive genetic effects, and ${\mathbf{e}}_{m}$ and ${\mathbf{e}}_{g}$ are vectors of residuals. The assumptions on the models are that $\mathbf{m}\sim N\left(0,{\sigma }_{m}^{2}\mathbf{M}\right)$, where $\mathbf{M}$ denotes the MRM and ${\sigma }_{m}^{2}$ is the variance due to the microbial effects, $\mathbf{g}\sim N\left(0,{\sigma }_{g}^{2}\mathbf{G}\right)$, where $\mathbf{G}$ denotes the GRM and ${\sigma }_{g}^{2}$ is the additive genetic variance, ${\mathbf{e}}_{m}\sim N\left(0,{\sigma }_{{e}_{m}}^{2}\mathbf{I}\right)$ and ${\mathbf{e}}_{g}\sim N\left(0,{\sigma }_{{e}_{g}}^{2}\mathbf{I}\right)$, where $\mathbf{I}$ denotes the identity matrix, ${\sigma }_{{e}_{m}}^{2}$ is the residual variance for the microbial model in Eq. (1), and ${\sigma }_{{e}_{g}}^{2}$ is the residual variance for the genetic model in Eq. (2). The fixed effects used in the models consisted of the fixed class for birth and rearing rank, the fixed class for age of dam (1, 2, 3+), and the covariate of birthday deviation from the mean birthdate of all animals born from the same flock and birth year (bdev). For the methane traits (CH_4_, CH_4_Ratio, or CO_2_), a contemporary group consisting of the combination of birth flock, birth year and sex was fitted as a fixed class. For the LW8 trait, the contemporary group consisting of the interaction between flock, birth year, weaning weight mob, mob for liveweight at 8 and 12 months, mob for bdev and sex was fitted as a fixed class. The heritability is then ${\sigma }_{g}^{2}/({\sigma }_{g}^{2}+{\sigma }_{{e}_{g}}^{2})$ and the microbiability is ${\sigma }_{m}^{2}/({\sigma }_{m}^{2}+{\sigma }_{{e}_{m}}^{2})$. The heritability and microbiability were estimated using all the animals in the dataset. The MRM was calculated using in-house bash and R scripts.

#### Trait prediction

The ability of RMC profiles and host genomics to predict trait values that are correlated with PAC methane traits was assessed for each of the four traits in this study. This required partitioning the dataset into two components; a training set to build the prediction model and a validation set to assess the performance of the model for predicting PAC traits relative to the measured trait values. Two approaches were used for the validation: (1) forward prediction (FP) and (2) cross-fold validation (CV). With FP, the training set consisted of all the animals that were born between 2014 and 2020 (n = 3524) and the validation set consisted of all the animals born in 2021 (n = 1061). The set of tags for the RF pipeline were determined using only the samples from the training set (e.g., a tag was retained if 25% of samples in the training set had at least one read). For the CV approach, the dataset was divided up into 11 subsets, where each subset consisted of several cohorts that, where possible, contained similar animals (i.e., same flock) and ranged in size from 324 to 524 animals. The reason for defining the subsets in this manner is to investigate the performance of the RMC profiles to predict the phenotypes across farms and breeding years as environmental factors (e.g., sampling date, farm practices, feed) are a major source of variation in the RMC profiles. A full breakdown of the different subsets is given in the supplementary materials (see Additional file 3, Table S1). The choice of 11 subsets was based on an approximately 90% training and 10% test split while keeping subsets as similar as possible in terms of animals included. Under the CV approach, 10 subsets were used as the training set and the remaining subset was the validation set, which was repeated 11 times with each subset being used as the validation set once. The set of tags for the RF pipeline for the CV approach was determined using all the samples in the dataset.

The prediction models used were of the same form as Eqs. (1) and (2) and were also fitted in ASReml v4.2 [53]. These models were fitted using the training dataset and predictions of the animals direct breeding values ($\widehat{\mathbf{g}}$) and the microbial values ($\widehat{\mathbf{m}}$) were made for the animals in the validation set. The prediction accuracy was computed as the Pearson correlation between $\widehat{\mathbf{g}}$ or $\widehat{\mathbf{m}}$ with the adjusted phenotype values (${\mathbf{y}}^{\mathbf{*}}$) for the animals in the validation set. The adjusted phenotype is the trait values adjusted for the fixed effects and is computed as the residuals from the linear model:

3 $${\mathbf{y}} = {\mathbf{Xb}} + {\mathbf{e}},$$

where ${\mathbf{y}}^{\boldsymbol{*}}=\boldsymbol{ }\mathbf{e}$. The slope of the adjusted phenotypes (${\mathbf{y}}^{\boldsymbol{*}}$) regressed against the predicted breeding and microbial values were also computed from the linear model of the adjusted phenotypes (${\mathbf{y}}^{\boldsymbol{*}}$) against $\widehat{\mathbf{g}}$ or $\widehat{\mathbf{m}}$.

#### Phenotypic and genetic correlations

A bivariate linear mixed model was fitted to assess the genetic and phenotypic correlations of the traits predicted from RMC profiles relative to direct measurements of the methane traits collected using PAC. The bivariate analysis was only run for the two methane traits CH_4_ and CH_4_Ratio, as RMC profiles have the greatest utility in rumen related traits [30]. To ensure an independent set of animals were used to mitigate against obtaining overly optimistic correlation estimates, only predictions from RMC profiles from the animals in the validation set were included in the bivariate analysis. This means that for the FP approach, phenotypic and genetic correlations were only estimated using the born 2021 animals that made up the validation set (n = 1053), whereas for the CV approach, phenotypic and genetic correlations were estimated using all the animals as there was a prediction for each animal (n = 4532$$)$$ since each fold had a turn at being the validation set. The bivariate model used for this analysis was:

4 $$\left[ {\begin{array}{*{20}c} {{\mathbf{y}}^{\user2{*}} } \\ {{\hat{\mathbf{m}}}} \\ \end{array} } \right] = \left[ {\begin{array}{*{20}c} {{\mathbf{Z}}_{1} } & \mathbf{0} \\ \mathbf{0} & {{\mathbf{Z}}_{2} } \\ \end{array} } \right]\left[ {\begin{array}{*{20}c} {{\mathbf{u}}_{1} } \\ {{\mathbf{u}}_{2} } \\ \end{array} } \right] + \left[ {\begin{array}{*{20}c} {{\mathbf{e}}_{1} } \\ {{\mathbf{e}}_{2} } \\ \end{array} } \right],$$

where ${\mathbf{y}}^{\mathbf{*}}$ is the adjusted phenotype defined earlier, $\widehat{\mathbf{m}}$ is the predicted microbial values and forms the proxy trait for $\mathbf{y}$ from the RMC profiles, ${\mathbf{Z}}_{1}$, ${\mathbf{u}}_{1}$ and ${\mathbf{e}}_{1}$ are respectively the incidence matrix for the random effects, the vector of breeding values and the vector of residual errors for the adjusted phenotype, ${\mathbf{Z}}_{2}$, ${\mathbf{u}}_{2}$ and ${\mathbf{e}}_{2}$ are respectively the incidence matrix for the random effects, the vector of breeding values and the vector of residual errors for the proxy RMC trait, and $\mathbf{0}$ denotes a rectangle matrix consisting of all zeros. The model assumes that $$\left[\begin{array}{c}{\mathbf{u}}_{1}\\ {\mathbf{u}}_{2}\end{array}\right]\sim MVN\left(\mathbf{0},\mathbf{G}\otimes \mathbf{B}\right),$$ where **G** is the GRM introduced earlier and $$\mathbf{B}=\left[\begin{array}{cc}{\sigma }_{{u}_{1}}^{2}& {\sigma }_{{u}_{12}}\\ {\sigma }_{{u}_{12}}& {\sigma }_{{u}_{2}}^{2}\end{array}\right]$$ is the variance–covariance matrix of additive breeding values, where ${\sigma }_{{u}_{1}}^{2}$ and ${\sigma }_{{u}_{2}}^{2}$ are the genetic variances for ${\mathbf{y}}^{\mathbf{*}}$ and $\widehat{\mathbf{m}}$ respectively and ${\sigma }_{{u}_{12}}$ is the genetic covariance between ${\mathbf{y}}^{\mathbf{*}}$ and $\widehat{\mathbf{m}}$. The model also assumes that $$\left[\begin{array}{c}{\mathbf{e}}_{1}\\ {\mathbf{e}}_{2}\end{array}\right]\sim MVN\left(\mathbf{0},\mathbf{I}\otimes \mathbf{C}\right)$$, where $$\mathbf{C}=\left[\begin{array}{cc}{\sigma }_{{e}_{1}}^{2}& {\sigma }_{{e}_{12}}\\ {\sigma }_{{e}_{12}}& {\sigma }_{{e}_{2}}^{2}\end{array}\right],$$ ${\sigma }_{{e}_{1}}^{2}$ and ${\sigma }_{{e}_{2}}^{2}$ are the residual variances for ${\mathbf{y}}^{\mathbf{*}}$ and $\widehat{\mathbf{m}}$ respectively and ${\sigma }_{{e}_{12}}$ is the covariance between residual terms. The phenotypic variances are ${{\sigma }_{p}}_{1}^{2}={\sigma }_{{u}_{1}}^{2}+{\sigma }_{{e}_{1}}^{2}$ for ${\mathbf{y}}^{\mathbf{*}}$ and ${{\sigma }_{p}}_{2}^{2}={\sigma }_{{u}_{2}}^{2}+{\sigma }_{{e}_{2}}^{2}$ for $\widehat{\mathbf{m}}$, and the genetic correlation is ${\rho }_{{u}_{12}}={\sigma }_{{u}_{12}}{\left({\sigma }_{{u}_{1}}^{2}{\sigma }_{{u}_{2}}^{2}\right)}^{-1/2}$. The heritability values are ${h}_{1}^{2}={\sigma }_{{u}_{1}}^{2}/{{\sigma }_{p}}_{1}^{2}$ for the adjusted PAC methane trait and ${h}_{2}^{2}={\sigma }_{{u}_{2}}^{2}/{{\sigma }_{p}}_{2}^{2}$ for the predicted microbial values of methane. All bivariate models were fitted using ASReml v4.2 [53] and standard errors were calculated using the delta method [53]. In addition, the CH_4_Ratio trait was converted into a percentage (multiplied by 100) when fitting the bivariate model to mitigate against numerical instability issues as the phenotypic variances were very small for this trait on the ratio scale.

## Results and discussion

### Genetic diversity

**Fig. 1:**
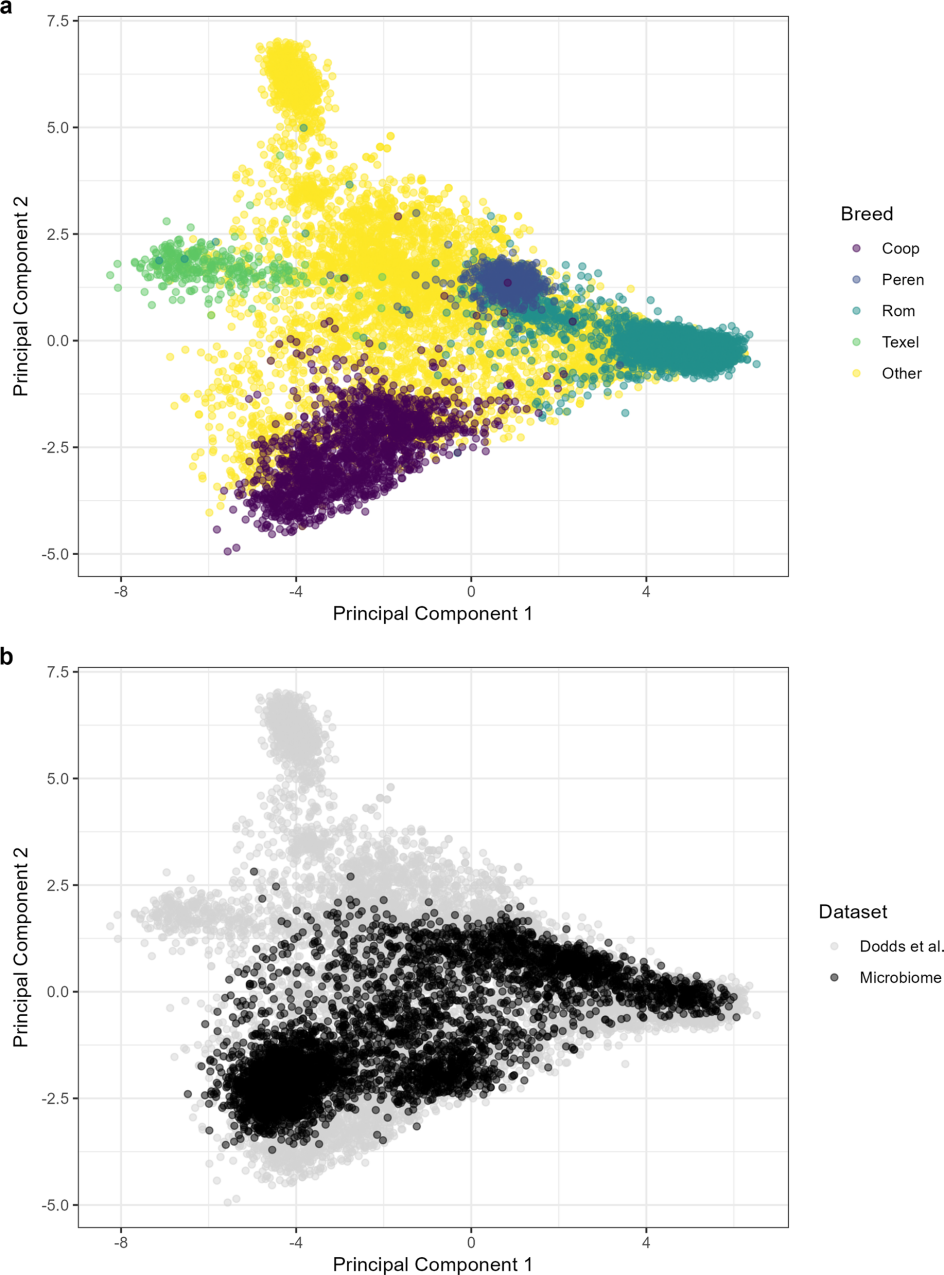
Principal component analysis (PCA) plot of the genomic relatedness matrix (GRM). The PCA analysis consists of the 4585 animals included in this study and 13,118 animals from the study by Dodds et al. [52]. **a** PCA plot of the Dodds et al. [52] animals coloured by breed type (Coop = Coopworth, Peren = Perendale, Rom = Romney). **b** PCA plot with the animals included in this study (black points) overlayed on the animals from the Dodds et al. [52] study (grey points)

The first two principal components of the combined GRM containing the animals in this study with the animals from the study by Dodds et al. [52] are shown in Fig. 1. The breed composition of the animals from Dodds et al. [52] shown in Fig. 1a, with PC1 separating Texel (negative PC1 values) from Perendale and Romney (positive PC1 values) and PC2 separating Coopworth (negative PC2 values) from these three breeds (PC2 values around zero or positive). A large proportion of the animals from the Dodds et al. [52] study were labelled ‘Other’ for breed composition. These animals were spread across and in-between all four named breeds on the PCA plots, suggesting a large number of composites were present. There were likely some other pure breeds present, with the group of ‘Other’ animals with high PC2 values likely consisting of breeds such as Primera, Suffolk or Poll Dorset [52].

Relative to these animals from Dodds et al. [52], the genetic diversity of the animals in the current study overlapped mostly with the Coopworth, Perendale and Romney animals, and also the composites that lie between these breeds (Fig. 1b). However, there were a few animals from the current study that were located towards but do not overlap with the Texel pure breeds, which suggests that some animals in the current study were composites containing Texel. Overall, these results suggest that the dataset used in this study covers a large proportion of the maternal genetics found in the New Zealand sheep industry.

### Heritability and microbiability

The heritability and microbiability estimates for the four traits in this study are given in Table 3. The heritability for the methane traits was 0.16 ± 0.021 for CH_4_, 0.21 ± 0.021 for CH_4_Ratio and 0.21 ± 0.022 for CO_2_. These estimates were similar to previous estimates found in the literature for methane traits measured using PAC on lambs grazing pasture [16]. For liveweight at 8 months (LW8), the heritability was 0.21 ± 0.023 which was lower than previous estimates found in the literature which are around 0.35 [30, 54]. The lower heritability estimate for LW8 could be due to pre-selection in the dataset as only animals that were rumen sampled were included and by the time rumen sampling for the industry flocks was performed, the lighter animals in the flock were likely already culled. Despite low heritability values (all at or below 0.21), it's noteworthy that these estimates were significantly different from zero (i.e., mean − 1.96 × standard error > 0 which gives an approximation for significance).

**Table 3:** Heritability and microbiability estimates (standard errors)

Trait	Heritability	Microbiability
LW8	0.21 (0.023)	0.45 (0.060)
CH_4_	0.16 (0.021)	0.54 (0.052)
CH_4_Ratio	0.21 (0.021)	0.64 (0.054)
CO_2_	0.21 (0.022)	0.18 (0.046)

The microbiability estimates were approximately 2 to 3 times larger than the heritability estimates for all traits, except for CO_2_ where the microbiability estimate was slightly lower. When compared to the results from Hess et al. [30], the microbiability estimate for LW8 was similar but lower for CH_4_ and CH_4_Ratio by 0.2. The microbiability for CO_2_ was also non-zero whereas Hess et al. [30] obtained a microbiability of around zero for CO_2_.

### Trait prediction

**Fig. 2:**
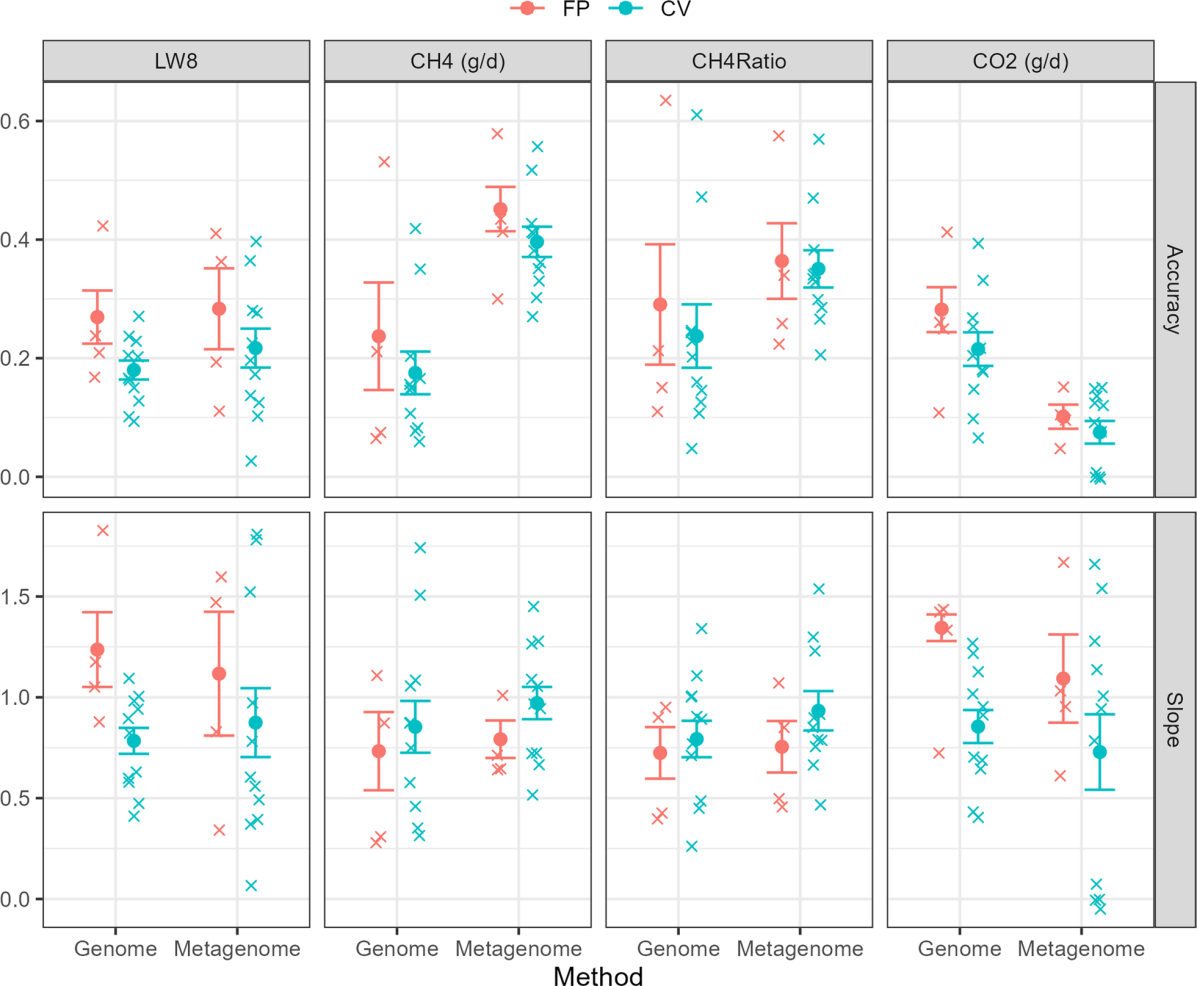
Results of predicting liveweight and methane traits using RMC profiles and host genomics. The predictive accuracy is given along the top row and regression slope is given along the bottom row of host genomics (Genome) and the RMC profiles (metagenome) for predicting the liveweight and methane traits in this study. Points and lines are coloured based on whether the statistics were computing using the forward prediction (FP) approach (red) or using the cross-fold validation (CV) approach (blue). Crosses denote the accuracy/slope for individual cohorts (FP) or folds (CV), circles denote the mean accuracy/slope and the error bars represent one standard error around the mean accuracy/slope (weighted by number of animals in cohort). Note that a value of 1 represents no bias in this context. The accuracy is calculated as the correlation between predicted and trait values adjusted for fixed effects

The accuracy and regression slope of predicting the traits using host genomics and the RMC profiles are shown in Fig. 2 (see Additional file 4, Table S2). The prediction accuracy and regression slope for LW8 was similar between host genomics and RMC profiles when compared within FP or CV approach, although the variation was larger for the RMC profiles. For the methane traits, the RMC profiles produced higher prediction accuracy compared to host genomics with a 1.9- to 2.3-fold increase for CH_4_ and 1.2- to 1.5-fold increase for CH_4_Ratio. The regression slope for CH_4_ and CH_4_Ratio were similar between using host genomics and RMC profiles, although it was slightly closer to 1 (i.e., less bias) for the RMC profiles. On the other hand, host genomics provided greater predictive accuracy for CO_2_ with over a twofold increase in accuracy compared to the RMC profiles. For some folds in the CV approach, the RMC profiles had little to no predictive ability (i.e., accuracy ≈ 0). Across all scenarios, the prediction accuracy was higher for the FP approach compared to the CV approach. This is not surprising since with the CV approach animals with similar genetics were placed in the same cohort and data from younger animals was used to train the model and predict trait values on their progenitors. This will lead to less genetic connectedness between cohorts and therefore less genetic connectedness between animals in the training and validation sets for the CV approach resulting in the lower prediction accuracies. The predictions from RMC profiles were checked for potential population stratification but none was observed (see Additional File 5, Fig. S3).

### Phenotypic and genetic correlations

Table 4 gives the genetic and phenotypic correlation estimates from the bivariate analysis for the validation animals for CH4 and CH4Ratio. For the FP approach, the genetic correlation between the methane measured using PAC and methane predicted from RMC profiles was moderate to high at 0.75 ± 0.12 for CH_4_ and 0.64 ± 0.11 for CH_4_Ratio, while the phenotypic correlations were lower but moderate at 0.40 ± 0.03 for CH_4_ and 0.31 ± 0.03 for CH_4_Ratio. These correlations were significantly greater than zero.

**Table 4:** Heritability, genetic and phenotypic correlations, and phenotypic variances from bivariate analysis of measured PAC and predicted RMC methane traits for validation animals

Trait	Setup	N^a^	Method	Heritability	Phenotypic variance	Phenotypic correlation	Genetic correlation
CH_4_	FP	1053	PAC^b^	0.21 (0.06)	2.27 (0.10)	0.40 (0.03)	0.75 (0.12)
			RMC^c^	0.37 (0.06)	0.66 (0.03)		
	CV	4532	PAC^b^	0.14 (0.02)	3.42 (0.07)	0.38 (0.01)	0.62 (0.07)
			RMC^c^	0.22 (0.02)	0.56 (0.01)		
CH_4_Ratio^d^	FP	1053	PAC^e^	0.34 (0.06)	0.45 (0.021)	0.31 (0.03)	0.64 (0.11)
			RMC^c^	0.42 (0.06)	0.098 (0.0047)		
	CV	4532	PAC^e^	0.20 (0.02)	0.53 (0.012)	0.33 (0.01)	0.55 (0.06)
			RMC^c^	0.22 (0.02)	0.077 (0.0017)		

The abbreviations are *PAC* Portable accumulation chamber, *RMC* Rumen metagenome community, *FP* forward prediction, *CV* cross-validation

^a^Number of animals in the validation set used in the bivariate analysis

^b^Scaled to a mean of 7.5

^c^Scaled to a mean of 0

^d^Converted to percentage for bivariate analysis

^e^Scaled to a mean of 3.2

The results from the CV approach were similar to those obtained from the FP approach, although the phenotypic and genetic correlation estimates were between 0.05 to 0.11 lower and the heritability estimates were between 0.07 and 0.2 lower for the CV approach compared to the FP approach. These results are not surprising given that there is less genetic connectedness between the training and validation sets in the CV approach compared to the FP approach as discussed earlier. Nevertheless, the genetic and phenotypic correlations were still moderate and significantly greater than zero.

Compared to the heritabilities in Table 3, the heritability estimates for the PAC methane traits from the bivariate analysis (${h}_{1}^{2}$) were similar for the CV approach but were larger for the FP approach by 0.05 (CH_4_) and 0.13 (CH_4_Ratio). Importantly, the heritability of the microbial values (the RMC methane trait; ${h}_{2}^{2}$) was larger than the heritability of the PAC methane trait (${h}_{1}^{2}$) in each bivariate model fitted, where the increase was between 0.02 and 0.16.

### RMC profiles as a proxy trait for PAC

In this study, we have investigated the potential of RMC profiles as a proxy trait for PAC methane emissions. As far as we are aware, this study is the largest to-date with a dataset of over 4500 sheep examining RMC profiles as a proxy phenotypic measure in livestock. Our results suggest that for the traits CH_4_ (g/d) and CH_4_/(CO_2_ + CH_4_) the RMC profiles provides better performance in terms of ranking an animal’s methane emissions relative to an overall set compared to using host genomics, which is in line with previous studies [30]. We also found for these traits that the genetic correlation between the RMC profile methane trait and PAC methane was moderate to high, and the RMC profile methane trait had higher genomic heritability estimates than the PAC methane trait. From our results, the relative efficacy (i.e., ${\rho }_{{u}_{12}}\sqrt{{h}_{2}^{2}}/\sqrt{{h}_{1}^{2}}$) of indirect selection via the RMC profile methane trait was 1 (FP) and 0.78 (CV) for ScCH_4_ and 0.71 (FP) and 0.58 (CV) for ScCH_4_Ratio. Jonker et al. [16] found a very high genetic correlation (> 0.93) of PAC methane on animals measured as lambs and again as adults. Hickey et al. [55] obtained repeatability estimates for PAC methane for animals measured as lambs and adults of 0.42 (CH_4_) and 0.34 (CH_4_Ratio). Furthermore, Hess et al. [30] found that RMC profiles collected on lambs were correlated with PAC methane measured at both the lamb and adult stages. Overall, these results suggest that the RMC methane trait is a suitable proxy for methane emissions measured using PAC and can be used for ranking animals based on their methane emissions for selective breeding. The dataset used in this study consists of animals from flocks across New Zealand, including central progeny test flocks used to provide genetic linkage across the industry, and broadly encompasses the maternal breeds found in the New Zealand population. This was supported by the fact that the animals in this study overlapped with a previously published dataset containing animals across the New Zealand maternal sheep [52] on the principal component analysis plot of a GRM containing both sets of animals. Therefore, the results from this study indicate that the RMC profile methane trait will be applicable at a national level.

Our results also suggest that RMC profiles cannot produce a reliable proxy trait for CO_2_ (g/d) phenotype due to poor performance of ranking an animal’s CO_2_ emissions relative to the overall flock. The liveweight trait LW8 was included in the analysis to provide a consistency check of the data and ensure that any apparent improvement in ranking animals from using RMC profiles was not due to an unusual characteristic of the genomic data. The heritability estimate for the LW8 trait were lower than previous studies [30, 54], which we assume is due to preselection, and the RMC gave similar performance to using host genomics. These results provide some reassurance that the data is reliable and the conclusions from this study are valid.

One limitation of this study is that only a single measure for RMC was collected on each animal and used for ranking. Collecting additional measures for each animal would likely improve accuracy and lead to faster genetic gains due to increased accuracy of the estimated breeding values for PAC methane. However, from a practical and animal ethics point of view, collecting a single RMC measure is preferred and is sufficient for RMC profile to be used a proxy measure of PAC methane traits.

### RMC profiles vs PAC for methane

As of 2024, the cost of taking a PAC measurement in New Zealand is $60NZD (up from $40NZD from 2 year ago) per animal. The cost of generating a RMC profile, from collection of the sample to sequencing to analysis, would be similar to a PAC measurement per animal. Although RMC profiles do not necessarily provide a cost benefit for obtaining methane measurements over PAC, there are several potential benefits of using RMC profiles instead of PAC. The first benefit relates to throughput and logistics. For a standard 8-h workday, the typical number of animals that can be rumen sampled with a team of two is approximately 250–300 (e.g., an average around one and a half to 2 min per animal), whereas for PAC the maximum number of animals for a single trailer would be 96 (equivalent to 8 lots). Furthermore, scaling up the animal numbers per day is more straightforward for RMC profiles as this would require training and deploying more technicians whereas more trailers would be needed for PAC, which requires having the underlining infrastructure in place that needs substantial up-front investment. PAC also faces logistical challenges in that PAC trailers need transporting between farms which can be costly and time consuming depending on the distance between farms.

An additional benefit of RMC profiles is that there is potential that it could be used for other difficult to measure traits. One such trait would be feed efficiency, where several studies have found that RMC profiles are predictive of various feed efficiency traits in ruminants [30, 56–60]. Obtaining feed intake/efficiency data in conjunction with methane measurements could be important for recognition of low methane emitting animals as regulatory bodies typically desire methane per kilogram of dry matter ingested to be in concordance with reporting standards for methane [21]. It should be noted that carbon dioxide emissions from PAC have been found to be a potential proxy for feed intake [61]. For other ruminants, the benefit of using RMC profiles to predict methane ranking over direct measures may be greater compared to sheep. For example, in cattle a suitable PAC protocol is still under development and obtaining methane measurements in paddocks is usually via the GreenFeed system or the sulphur hexafluoride tracer method meaning that it is more difficult and expensive to measure methane using existing methods compared to sheep. On the other hand, the cost of generating a RMC profile in cattle will be similar to sheep, but with the extra challenge being collecting the rumen sample, which is more difficult in these bigger animals. However, most NZ cattle bull breeding operations have installed cattle weigh crushes with head bails and backing bars which are suitable for collecting rumen samples with minimal alteration.

### Practical considerations for RMC profiles

There are several challenges with using RMC profiles as a tool for phenotyping. The primary challenge is that the main source of variation in RMC profiles is due to environment variables (e.g., time of sampling, feed type). For the analysis conducted in this study, the animals within a cohort were similar (i.e., same farm, same sex, same birth year, sampled within a few days, and grazed pasture under similar conditions), but environmental variation between cohorts due to differences in sampling time and location is present. This environmental variation can weaken the signal in the RMC profiles for the trait of interest resulting in poor predictive performance. To overcome this issue, cohort standardization is used when constructing the MRM to enable more robust rankings when predicting the genetic merit of an animal’s methane emissions across farms and breeding years. Furthermore, to reduce variation between cohorts, all cohorts were kept consistent such that each cohort consisted of animals that were lambs and grazing pasture. Although it has been shown that RMC profiles are predictive of methane ranking across diets and age [30], it is unclear whether cohorts with different feeds or ages could be combined into the same analysis to produce a reliable proxy trait for PAC methane that is still genetically correlated. At present, our recommendation is to use cohorts consisting of animals that are broadly the same age and consuming the same type of feed to generate an RMC proxy methane trait while additional information is collected.

Another unwanted source of variation with RMC profiles is laboratory effects. In our experience, most laboratory effects (i.e., plate or well effects) contribute very minor variation on the RMC profiles [23]. However, we have found that there are non-trivial amounts of variation in the RMC profiles due to running the same samples on different flowcells. Consequently, the cohort standardized method used to construct the MRM needs to account for sequencing flowcell such that samples within a cohort must have been sequenced on the same run. To maximise cohort sizes, it is therefore advisable when designing plate layouts for sequencing that samples from the same cohort are placed on plates that are sequenced on the same flowcell run. For commercial applications, it would therefore be more practical to charge breeders or farmers in batches of 46 or 92 samples for RMC profiling (allowing for 2 positive and 2 negative controls on a 96-well plate) as this would avoid issues with trying to fill libraries without splitting cohorts across flowcells.

### Implementing RMC profiles in breeding program

To implement RMC profiles from RE-RRS as a proxy phenotype for PAC methane in a commercial breeding scheme would require overcoming several challenges. The first is developing a method that incorporates the RMC profile methane phenotype into a national genetic evaluation system. This is challenging as animals in a genetic evaluation will have different levels of information (i.e., pedigree, genotypes, RMC profiles, methane traits) available. One solution would be to use a 2-step approach whereby PAC methane emissions are predicted from RMC profiles to produce a proxy trait which is then incorporated in the model that is used to run a national genetic evaluation, similar to the bivariate model used in this study. This would allow estimation of methane breeding values for all individuals with pedigree or genotype data, regardless of whether RMC profiles and/or methane traits have been collected or not.

The second challenge is having a method that can generate the RMC profile methane trait in the sufficiently short timeframe that would be required for a genetic evaluation. For the analysis method we have utilized, the biggest bottleneck in computation is generating the MRM. We utilised a RF bioinformatics pipeline to generate an RMC profile that was used to construct the MRM. The RF pipeline has the advantage of being relatively fast and with low memory requirements but typically produces an RMC profile that is high-dimensional (> 200,000 tags) with sparse counts. One approach to reduce the dimension would be to align the tags to a reference database, which would effectively bin tags by taxonomy. However, reference databases are usually biased and missing potentially important microbes that have not been cultured or sequenced, which could provide important information for methane traits. Reference databases are also updated regularly with changes in taxonomy classification frequently occurring, which would make performing a genetic evaluation using updated versions of a reference database difficult after the RMC profile has been incorporated into the genetic evaluation without requiring re-running the entire training dataset. An alternative solution would be to generate a virtual tag catalogue that captures a sufficient number of important sequences from microbes that are present in rumens of sheep grazing pasture. This would reduce computation time as the set of tags used in the RF pipeline would not need regenerating each time the training set was increased with more samples and would prohibit the size of the tag set growing exponentially to the point where the computation would become unwieldy. However, the minimum number of tags that can be used while maintaining ranking accuracy and genetic correlation with direct measurements would need to be investigated. In addition, clustering tags into meaningful groups could be another solution to reducing the dimensionality of the RF RMC profiles, although an appropriate way to cluster tags and the impact on predictive would need to be explored.

One advantage of the 2-step approach we have proposed above is that it allows flexibility around the method used to generate the RMC rankings of PAC methane compared to a model where the genomics and RMC information is combined to generate PAC methane breeding values. This would allow new methods for predicting PAC methane from RMC profiles that are developed at a later date to be used in the breeding program, where these new methods could provide better solutions to the challenges faced with the approach we have used in this study. This also applies to alternative proxies, such as obtaining RMC profiles from buccal (i.e., oral) swabs and the development of spectral capture of metabolites in milk and meat samples. Some of these alternative proxies are the focus of current research and once developed could be incorporated into existing breeding programs in similar manner as discussed here.

One important consideration for implementing a breeding program would be the reference population used for training the prediction model. The training population would require animals with both PAC and RMC measurements. In practice, breeders and farmers would only collect one measurement type to generate the methane breeding values they require, and we would expect both PAC and RMC profiles to be used if RMC profile were available commercially. One strategy for building a reference population would be to use the current dataset as an initial reference population and then add additional data by collecting PAC and rumen samples from future generations of the national progeny test flocks. As the progeny test flocks provide genetic linkage across the industry in New Zealand, we would aim to collect 400–500 measurements to maintain the reference population. Another consideration is which breeding value(s) are used for methane in the selection index when using RMC profiles. Under the 2-step approach discussed above, we would propose using the PAC methane breeding values computed from the bivariate model that incorporates the RMC information as the methane breeding value used in the selection index. This would be feasible to implement and would allow other proxy measures of PAC methane to be included in the future. In addition, the PAC methane trait is already included in the current selection index and its genetic correlations with other traits have already been estimated [55].

## Conclusions

Breeding for low methane emitting animals to help meet national and global methane reduction targets requires obtaining large-scale methane emission measurements in a national herd. In this study, we have utilized a low-cost, high-throughput method to generate rumen metagenome community (RMC) profiles from rumen samples taken in a large number of sheep across New Zealand, and these were used to produce a proxy trait for various PAC methane traits. We have shown that the methane traits generated from the RMC profiles are predictive of an animal’s methane emissions relative to an overall flock and are genetically correlated with PAC methane traits. Therefore, RMC profiles are a suitable proxy for methane traits measured using PAC. RMC profiles provide sheep breeders with an alternative method for ranking animals on their methane emissions in their flocks and so facilitate breeding for low-methane sheep. This study highlights the potential of RMC profiles in breeding for low methane emitting animals and could be applied in other ruminants such cattle where the financial benefit would be greater compared to sheep.

## Supplementary Information

Additional file 1: Figure S1. Principal component analysis (PCA) plot of the microbial relatedness matrix (MRM) generated from the cross-validation (CV) approach.

Additional file 2: Figure S2. Comparison of generating a MRM using centred log-ratio transformation (CLR) with generating an MRM using the log10 transformation.

Additional file 3: Table S1. Description of the subsets used for cross-validation (CV) prediction.

Additional file 4: Table S2. Accuracy and regression slope of predicting liveweight and PAC methane traits from host genomics and the RMC profiles.

Additional file 5: Figure S3. Predicted PAC methane from RMC profiles vs adjusted methane from PAC colored by main breed type for the forward prediction (FP) analysis.

Additional file 6: Table S3. Rumen sample metadata.

## Data Availability

The sequencing data analysed during the current study that was generated as part of a study reported by Hess et al. [23] is available in the NCBI Short Read Archive (SRA) database under BioProject ID PRJNA859547. Raw sequence data for the remaining samples are available in the NCBI Short Read Archive (SRA) database under BioProject ID PRJNA1106541. The phenotypic and genotypic data analysed during the current study are available from Sheep Improvement Limited or AgResearch, but restrictions apply to the availability of these data, which were used under license for the current study, and so are not publicly available. Metadata for the rumen samples and IDs used in the SRA database to link samples are available in supplementary materials (see Additional file 6, Table S3).
