# Supplementary material for: Rumen metagenome profiles are heritable and rank the New Zealand national sheep flock for enteric methane emissions
Source: Genet Sel Evol. 2025 May 27;57:25. doi: 10.1186/s12711-025-00973-3 (PMC12117806; doi:10.1186/s12711-025-00973-3)
Supplement: Supplementary file 1 — Additional file 1: Figure S1. Principal component analysis (PCA) plot of the microbial relatedness matrix (MRM) generated from the cross-validation (CV) approach. [file 12711_2025_973_MOESM1_ESM.docx]

Additional file 1


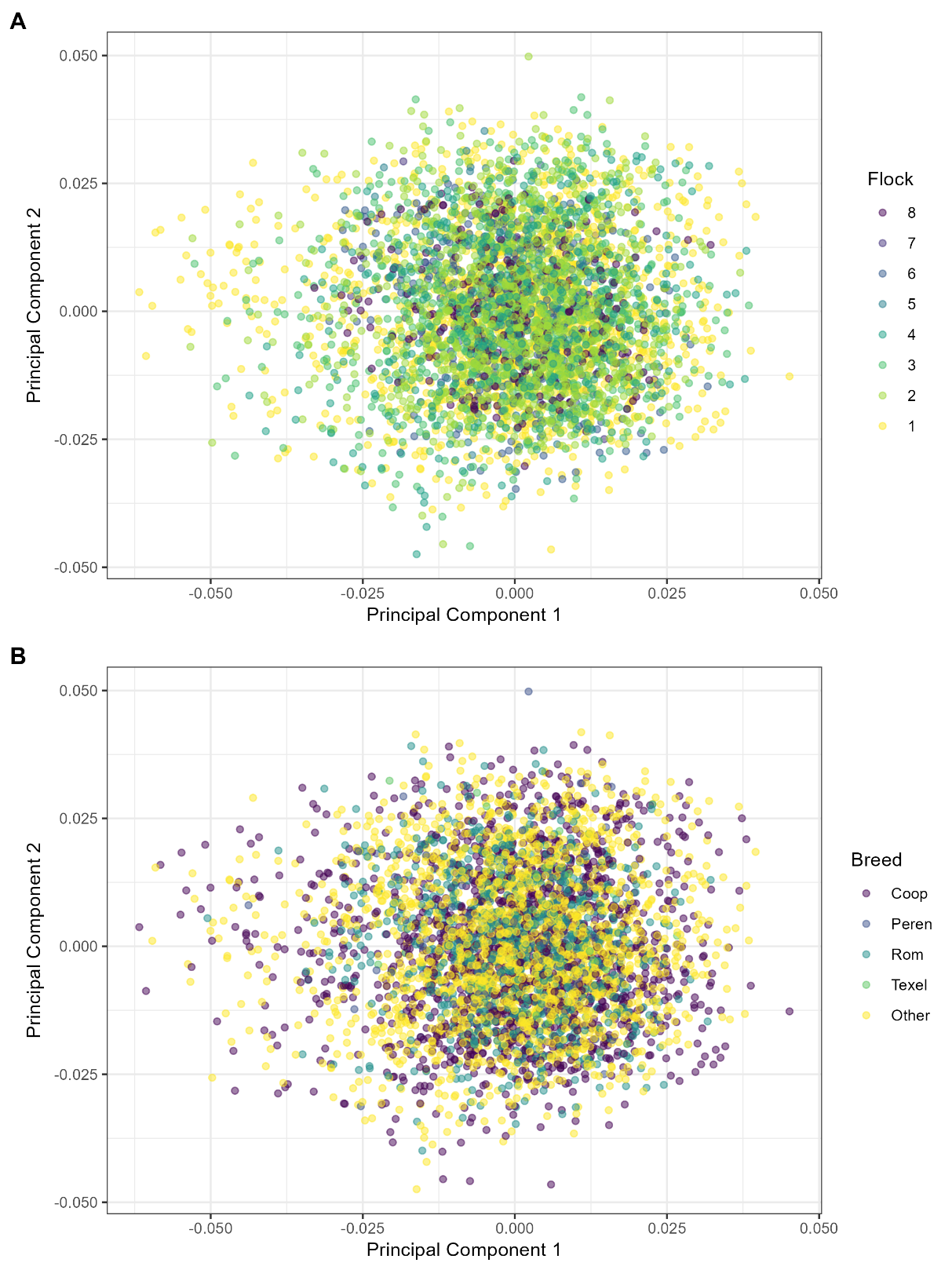


Figure S1: Principal component analysis (PCA) plot of the microbial relatedness matrix (MRM) generated from the cross-validation (CV) approach, and coloured by (a) flock and (b) breed type. The breed groups are Coopworth (Coop), Perendale (Paren), Romney (Rom), Texal, and composition, crossbreeds and other main breed types (Other). An animal was assigned a main breed type if 50% or more of that breed, otherwise the animal was given the breed type Other.
