# Supplementary material for: Rumen metagenome profiles are heritable and rank the New Zealand national sheep flock for enteric methane emissions
Source: Genet Sel Evol. 2025 May 27;57:25. doi: 10.1186/s12711-025-00973-3 (PMC12117806; doi:10.1186/s12711-025-00973-3)
Supplement: Supplementary file 2 — Additional file 2: Figure S2. Comparison of generating a MRM using centred log-ratio transformation (CLR) with generating an MRM using the log10 transformation. [file 12711_2025_973_MOESM2_ESM.docx]

Additional file 2


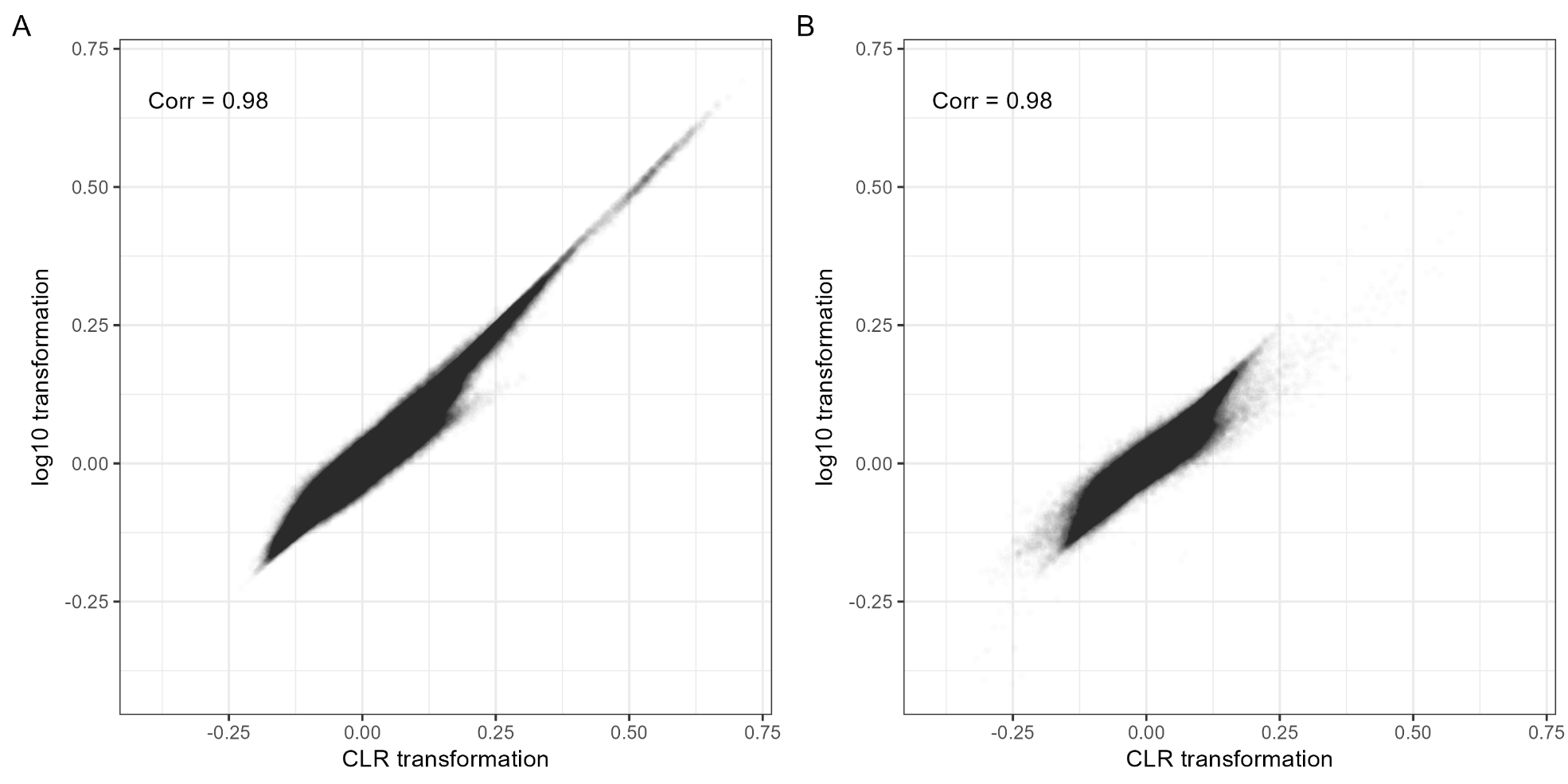


Figure S2: Comparison of generating a MRM using a centred log-ratio transformation (CLR) compared to generating an MRM using the log10 transformation used in this study for (A) no cohort adjustment and (B) with cohort adjustment. The two methods used the same approach to generate the MRM, except that the CLR transformation was used (and computed using the mixOmics package (via the logratio.transfo function)) for step two.
