# Supplementary material for: Rumen metagenome profiles are heritable and rank the New Zealand national sheep flock for enteric methane emissions
Source: Genet Sel Evol. 2025 May 27;57:25. doi: 10.1186/s12711-025-00973-3 (PMC12117806; doi:10.1186/s12711-025-00973-3)
Supplement: Supplementary file 5 — Additional file 5: Figure S3. Predicted PAC methane from RMC profiles vs adjusted methane from PAC colored by main breed type for the forward prediction (FP) analysis. [file 12711_2025_973_MOESM5_ESM.docx]

Additional file 5


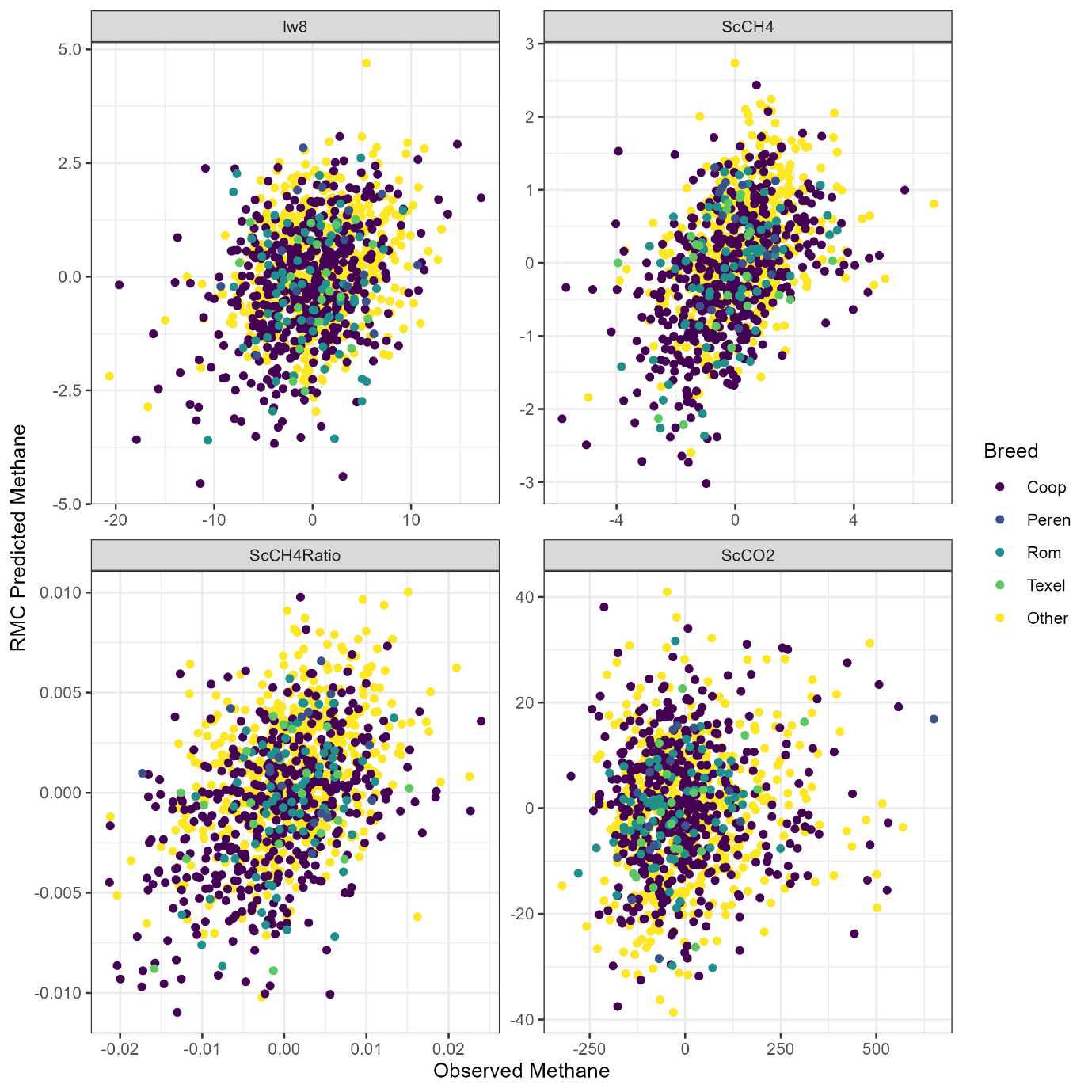


Figure S3: Predicted methane from RMC profiles vs adjusted methane from PAC colored by main breed type for the four traits analyzed in this study. The breed groups are Coopworth (Coop), Perendale (Paren), Romney (Rom), Texal, and composition, crossbreeds and other main breed types (Other). An animal was assigned a main breed type if 50% or more of that breed, otherwise the animal was given the breed type Other.
